# Use of Chia by-Products Obtained from the Extraction of Seeds Oil for the Development of New Biodegradable Films for the Agri-Food Industry

**DOI:** 10.3390/foods10030620

**Published:** 2021-03-15

**Authors:** Nuria Muñoz-Tebar, Ana Molina, Manuel Carmona, María Isabel Berruga

**Affiliations:** Food Quality Research Group, Institute for Regional Development (IDR), Universidad de Castilla-La Mancha, 02071 Albacete, Spain; nuria.munoz@uclm.es (N.M.-T.); ana.molina@uclm.es (A.M.); manuel.carmona@uclm.es (M.C.)

**Keywords:** chia seed by-products, defatted chia flour mucilage, biodegradable, edible coatings

## Abstract

Chia oil production and consumption have increased in recent years, producing a large number of by-products that had low utility or economic value for the industry. In this study, a biodegradable film was successfully prepared from mucilage extracted from defatted chia flour. The physical-chemical, optical, water vapor permeability (WVP), and mechanical properties of films made with two different types of chia matrixes (defatted flour and whole seeds) were determined. In general, defatted chia flour films exhibited a slightly reddish and yellowish color but still transparent in appearance, were good visible light barriers, and had better mechanical properties than films made with whole seeds. They also have greater WVP values than synthetic films such as low-density polyethylene. The results of the present study demonstrated that defatted chia flour can be used in producing edible films with improved quality characteristics.

## 1. Introduction

In the last 50 years, the food industry has used a diverse range of synthetic petroleum-based plastics [1] that are non-renewable and non-biodegradable, becoming a major environmental problem that concerns the consumers and all participants of the food production chain [1]. Europe generated 174 kg per inhabitant of packaging waste in 2018 and 19% corresponds to plastic material [2]. For this reason, the current consumer demands more natural foods, free of synthetic compounds in addition to environmentally friendly food packages. These claims have caused companies and researchers to collaborate to offer improved and healthier products [3]. In this sense, one of the main trends consists in the development of innovative films obtained from agricultural or food by-products, as well as renewable natural resources, as an effective and economical replacement for conventional plastics [4].

The use of edible films based on natural polymers has emerged as an alternative to plastics and as a solution for the pollution issue that has increased in the last few years. In addition, the edible films and coatings can be used as carriers for antioxidants or antimicrobials additives to extend food shelf life [3] and once combined with the biopolymers might lead to new film properties [5]. The edible polymers can be classified according to their origin into two types: hydrocolloids (polysaccharides and proteins) and lipids (fatty acids and waxes, resins and composites) [6]. Among them, it can be highlighted plant seed polysaccharides constitute one of the most important hydrocolloids used in the food industry because of their technological properties and dietary characteristics [7]. Polysaccharide-based films are usually based on cellulose, alginates, gums, chitosan, and recently mucilage, and the main reason why they are used in the development of new biofilms is that they can be metabolized by the human body along with food, turning them into edible films [8].

In this context, chia seeds (*Salvia hispanica* L.) and its products like oil (source of α-linolenic acid and natural antioxidants), or by-products such as residual flour (source of insoluble dietary fiber and protein gluten-free) and mucilage (soluble dietary fiber), fulfill the requirements for edible films formation. The functional properties of the chia mucilage indicate that it is a polymer with a thickening property, a high viscosity in water, and possible beneficial metabolic effects. Chia mucilage is mainly composed of carbohydrates with 75–78%, followed by proteins with 9.7–10.6% [9,10], ash with 8.79%, and lipids with 2% [10]. Likewise, it is an anionic heteropolysaccharide composed of xylose and glucose in a 2:1 ratio, with uronic acids (glucuronic and galacturonic acids [11] that can be extracted from the seeds in an aqueous extraction [12]. Furthermore, it has great potential for use in the food industries as a stabilizer, emulsifier, thickener, additive or fat substitute [13,14,15] due to its slimy nature at low concentrations [16].

To the best of our knowledge, there are no studies so far on films made with mucilage extracted from defatted chia flour. The objective of this work was to develop and evaluate the physical, optical, and mechanical properties of films from chia by-products and compare them with films made using the mucilage extracted from whole seeds. The goal is to revalue this by-product and promote the circular economy in the food industry.

## 2. Materials and Methods

### 2.1. Materials

Commercial organic chia seeds (Biogran S.L., Madrid, Spain) were purchased and had a chemical composition (g/100 g) of 31.1 fat, 21.2 protein, 3.8 saturated fatty acids (SFAs), and 17.8 of α-linolenic acid. Glycerol (analytical grade) and Tween 20 (food grade) were obtained from Guinama S.L.U. (Valencia, Spain) and Sigma-Aldrich (St. Louis, MO, USA), respectively.

### 2.2. Defatting of Chia Seeds and Mucilage Extraction

The oil was obtained from the chia seeds (*Salvia hispanica* L.) in a hydraulic press (MECAMAQ Model DEVF 80, Vila-Sana, Lleida, Spain) as described by Muñoz-Tebar et al. [17]. The defatted chia flour was grounded at 10,000 rpm for 3 min with a knife mill GRINDOMIX GM 200 (Retsch, Haan, Germany) and stored vacuum-packed in darkness at 4 °C until use. The chia mucilage (CM) was extracted from defatted flour and chia nutlets according to Muñoz et al. [12] with slight modifications. Ten grams of sample (defatted flour or chia nutlets) were placed in an Erlenmeyer flask, distilled water was added at a ratio 1:40 (sample: water), and the pH was adjusted at 8 with 0.1 M NaOH. The extraction was performed for 2 h at 80 ± 2 °C under constant stirring and the solution was centrifuged (Beckman Ultracentrifuge, CA, USA) at 10,000 rpm, 30 min, 20 °C. Then, the mucilaginous suspension was filtered through a gauze, spread on a tray, and dried at 50 °C overnight. Finally, the dried mucilage was recovered from the tray, weighed to calculate the extraction yield, and stored in a desiccator until use.

### 2.3. Preparation of the Chia Films

The chia films were prepared based on the procedure described by Dick et al. [18], with slight modifications. Briefly, dried CM (1.5% *w*/*v*) was dissolved in distilled water, the pH was adjusted to 9 with 0.1 M NaOH and stirred at 25 °C for 3 h to obtain a homogeneous solution. The solution was then heated at 80 ± 2 °C for 30 min under stirring and 35% of glycerol and 15% of Tween 20 (*w*/*w* based on CM weight) were added. After heating, the film solution was stirred for 30 min at room temperature, cast onto petri dishes (0.55 g/cm^2^), and placed in an oven with air convection (Heraeus, Hanau, Germany) at 35 ± 1 °C overnight. Then, the films were peeled and stored in a desiccator at 25 °C and 52% RH for at least 48 h before their characterization.

### 2.4. Characterization of Chia Biofilms

#### 2.4.1. Moisture Content and Water Solubility (WS)

The determination of moisture content and water solubility of the films was carried out according to Dick et al. [18] with minor modifications. For moisture, film samples of 2 cm diameter were dried at 105 °C in an oven (J.P. Selecta, Barcelona, Spain), and the moisture content was calculated in triplicate gravimetrically after 24 h of drying. The water solubility of the films was determined with the dried films from the moisture analysis, defined as the initial dry weight (*Wi*), which were immersed in 30 mL of distilled water and stirred in an orbital shaker (OVAN, Barcelona, Spain) at 150 rpm and 22 °C for 24 h. Subsequently, the samples were filtered with a pre-weighed desiccated filter paper and the undissolved fractions of the film were dried in an oven at 105 °C for 24 h. The resulting dried material was weighed (*Wf*) and the water solubility was calculated in triplicate using the following equation:

*WS* (%) = [(*Wi* − *Wf*)/*Wi*] × 100 (1)

#### 2.4.2. Water Vapor Permeability (WVP)

The water vapor permeability of the films was determined in triplicate based on Dick et al. [18], with slight modifications. Chia films (55 mm diameter circles) were sealed on permeation cells (1.22 × 10^−2^ m^2^) containing 50 mL of distilled water and placed inside a desiccator with silica gel (0% HR, 24 °C). Samples were weighed during 10 h at 2 h intervals to monitor the weight loss over time. Finally, the water vapor permeability (WVP) of chia films was calculated with the following equation:

(2) $$WVP\;\left({g\;s}^{-1}m^{-1}{\;\mathrm{Pa}}^{-1}\right)=\frac{w\;\times \;L}{A\;\times \;t\;\times \;\Delta P}$$

where *w* is the weight of the water that permeated through the film (g), *L* is the film thickness (m), *A* is the permeation area of the cell (m^2^), *t* is the time of permeation (s), and Δ*P* is the water vapor partial difference (Pa) across the two sides of the film.

#### 2.4.3. Color and Opacity

The color of the films was measured in the reflection mode with a Minolta CR-400 colorimeter (Minolta, Japan) with a CR-A33a cone, a D65 illuminant, and an angle vision of 10°, following the method described by Costa et al. [19]. For calibration, a white standard color plate (Y = 93.1, x = 0.3160, and y = 0.3323) was used and the L* a* b* coordinates were measured in triplicate. The opacity of the films was calculated in triplicate according to the Hunter Lab method with the following equation: Opacity (%) = (Yb/Yw) × 100, where Yb is the opacity on a black standard and Yw is the opacity on a white standard. The total color difference (∆E) was calculated according to Dick et al. [18] with a white standard plate (L* = 97.24, a* = 0.09, and b* = 1.86), and the whiteness index (WI) of the films was measured using the method and equation described by Khazaei et al. [20].

#### 2.4.4. Light Transmittance

The capacity of light transmittance of chia films was measured according to Dick et al. [18] with minor modifications. Chia films were cut into rectangles (4 × 0.8 cm) and placed in a spectrophotometer cell. The transmittance was measured by spectrum scanning (wavelengths from 200 to 850 nm) with a spectrophotometer (Spectronic Helios α UV-Vis, Thermo). Air was used as a reference and the transmittance values (expressed as % of transmittance) were measured in triplicate.

#### 2.4.5. Thickness and Mechanical Properties

The thickness of the films (mm) was measured in triplicate at three different points for each sample using a digital micrometer IP65 Coolant-Proof (Mitutoyo, Japan) with a precision of ±0.01 mm. Mechanical properties of chia films were determined according to the procedure described in the standard ASTM D882-10 [21] in a texturometer TA-XT2i (Stable Micro Systems Ltd., Godalming, Surrey, UK). For that, samples (2 × 10 cm) were placed between the A/MTG tensile grips with an initial distance of 80 mm, and the force and deformation were recorded at a speed of 0.00083 m/s. Nine strips from each film were measured and tensile strength (TS) and elongation-at-break were expressed in MPa and % elongation, respectively.

### 2.5. Statistical Analysis

Statistical analysis of data was performed using SPSS (IBM SPSS Statistics version 25). ANOVA (one way) was calculated using a confidence level of 95% to determine any significant difference between the films from defatted chia flour and films made with whole seeds.

## 3. Results

### 3.1. Mucilage Extraction Yields

The mucilage extraction yield of the chia was significantly higher (*p* < 0.001) when we used defatted flour instead of the whole seeds. The results of the extraction yield were 15.3 ± 0.4% for the defatted flour and 6.6 ± 0.2% for the seeds, which meant that almost 60% more chia mucilage was obtained from defatted chia flour. The difference in yield may have been due to the variability of the mucilage composition, as well as the compounds released after ground the seeds, such as proteins. These changes in the composition of chia mucilage before and after defatting have already been observed by other authors [22], who found that partially defatted chia gum contained 33% more protein than fatted chia gum.

Moreover, the yield value of the seeds was similar to those reported by Muñoz et al. [12].

### 3.2. Moisture Content, Water Solubility, and Thickness

The moisture content of the films made from different chia matrixes is summarized in Table 1. A fixed level of plasticizer was established to compare the moisture content of both films because it has been previously proved that the concentration and type of plasticizer affect the moisture content [20,23,24].

It was observed that the moisture of the film’s changes (*p* < 0.05) when we use mucilage obtained from different chia matrixes (Table 1). This could mean that the defatting process affected the moisture content of the films by reducing it. A reduction in moisture content was also observed by Dick et al. [23] when they used chia flour instead of whole seeds.

Moreover, comparable results to those described by other authors for similar levels of glycerol in films made with chia [18] and quince [24] seeds were obtained.

The water solubility of the films is an important factor because it allows us to understand the behavior that the films will present when they come in contact with water, and in cases that the coating will be consumed along with the food, as well as for use in edible and biodegradable packaging that may require high solubility of the films [25]. The high-water solubility of both types of films proves their biodegradability and corroborates that both could be suitable for applications in packaging wrap. The data presented in Table 1 regarding water solubility shows that the values were significantly higher (*p* < 0.001) in the films made with whole seeds than in the films made with defatted flour. The solubility of the films made with whole seeds was slightly higher than the values obtained by Dick et al. [18] in films also made with whole chia seeds and this difference may be attributed to the different concentrations of mucilage used in the films (1.5 vs. 1%). Likewise, when comparing with other types of films, the use of chia mucilage (regardless of the matrix) provides higher values than basil seed films [20] and chitosan films [26] but in the same range as films made with sage seed gum [27] and mucilage from *Opuntia ficus-indica* [25].

Finally, thickness values of the films (Table 1) ranged from 0.09 to 0.12 mm, being significantly higher (*p* < 0.001) in the films made with defatted chia flour, and these differences may be attributed to the variability in the matrix (defatted flour and whole seeds) used in the film-making. The films formed with defatted flour showed lower thickness than films made with chia flour by Dick et al. [23] due to the different amounts of CM used in the film formation. The thickness of films made with whole seeds was consistent with those obtained by Muñoz et al. [28] and Salazar Vega et al. [29] in films made with whole chia seeds. Moreover, comparing the thickness of both films with other polysaccharide films, it was noticed that the values were slightly higher (0.09–0.012 vs. 0.07–0.08 mm) than films made with cress seed gum [30], psyllium seed [31], Balangu seed mucilage [32], and chitosan [26]. Thickness determines the technological properties of the films, so its control plays an important role. It has been observed that the main factors modifying film thickness are the amount of plasticizer added to the film [18] and the amount of coating cast [23,29]. Likewise, for its industrial application as a coatings/film, the factors to consider are the immersion time and the number of times the food is immersed.

### 3.3. Water Vapor Permeability (WVP)

The water vapor permeability plays a key role in the development of new edible coating due to it regulates water transfer between the coating and the external environment and this is a decisive factor in food spoilage. Therefore low WVP values are desirable [25,33]. As observed in Table 1, the WVP values were significantly higher (*p* < 0.001) in the films made with defatted flour than in the films made with whole seeds (0.58 vs. 0.33 × 10^−1^ g s^−1^ m^−1^ Pa^−1^). The lower water vapor permeability of the whole seeds films could be due to several factors: the lipid-protein interaction that causes more hydrophobic zones preventing the diffusion of water vapor through the film [34], the lower amount of protein and nitrogen-free extract in the fatted chia seed mucilage [22], and the possible release of other extracellular substances after grinding the seeds. Dick et al. [18,23] observed a 43% increase in the WVP values of the films when using chia flour instead of ungrounded seeds, which may be related to the release of extracellular components such as proteins and polysaccharides. In this sense, Salazar Vega et al. [29] observed an increase in protein and nitrogen-free (NFE) content of 33 and 50%, respectively, when chia for gum production was partially defatted.

When comparing the results to other films, it was observed that the WVP of the defatted flour films was comparable to the results obtained in chia seeds films by Dick et al. [18] and in films made with glucomannan by Kurt and Kahyaoglu [33], as well as that they are better water vapor barriers than films made with cress seeds [30], basil seeds films [20], and films made with balangu seeds [32]. The fact that both films (defatted flour and whole seeds) had lower WVP values proved that they were a great barrier to water vapor [27,35] than other films made with seeds. These properties could be useful for developing coatings for foods that require a long period of storage such as cheese or dry food like a bakery in which it is important to avoid rapid water permeation.

### 3.4. Color

Color is a key factor in consumer acceptance, so it is an important parameter that must be considered in the development of new edible coatings. As Table 2 shows, there were no differences in L* and b* coordinates between the two films and it happened to be the same total color difference (∆E) and whiteness index (WI). On the contrary, significant differences (*p* < 0.001) were observed in the coordinate a*. This coordinate is the red-green index so the highest values in coordinate a* (8.92 vs. 5.45) of whole seed films indicated that they were more reddish than films made with defatted flour, but these differences were not appreciated by the human eye.

Overall, the films were less bright than other chia seed films [18], basil seed films [20], sage seed films [27], and chitosan films [26], and the coordinates a* and b* indicated that the films were yellowish and reddish than other types of edible films made with polysaccharides [24,32,36]. Brighter and lighter colors in films might indicate that they can be used in packaging in which the content must be seen [29]. In this sense, the optical properties of both types of film prove that they could be a good alternative for coating bakery products or fruits where it is important to see the natural color of the product. Moreover, their softer color than the brown, yellow, or black colors of many polyvinyl acetate coatings used in many pressed hard cheeses such as Manchego or Gouda indicated that they could be a suitable alternative to be applied as coatings on cheeses.

### 3.5. Optical Properties

The films capacity of light transmittance and opacity are two important parameters to be evaluated in the development of new coatings because exposure to light visible and UV could cause oxidative deterioration of food products leading to nutrient losses, changes in color, and unpleasant flavors [5]. The opacity of the films is presented in Table 2, and a slight but significant difference (*p* < 0.01) was observed between the two films (25.21 vs. 26.87%). These results were consistent with the differences found in thickness since an increase in thickness leads to an increase in the opacity of the films [37].

The opacity of the films made with chia (regardless of the matrix) was higher than the values achieved by Costa et al. [19] and Ortiz de Elguea-Culebras et al. [26] in chitosan films (6.10 and 5.3%, respectively) and by Chambi and Grosso [38] in films made with polysaccharides/gelatin (12.4–12.7%). The films developed in the present work were not very opaque and visually transparent, which made them suitable to be used as food coating or for improving product appearance.

**Figure 1:**
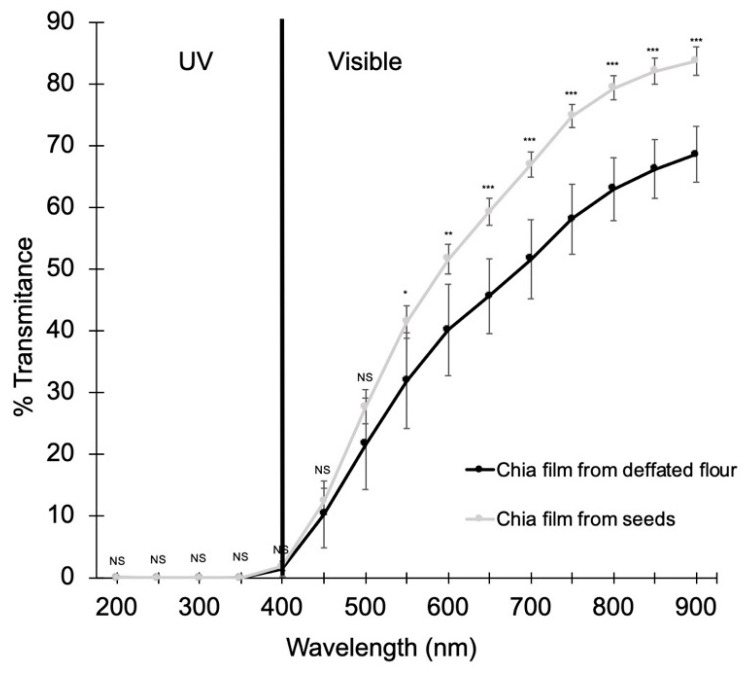
Light transmittance capacity of the films made with whole seeds and defatted flour. Significance differences are indicated as follows: NS: Not significant, * *p* < 0.05, ** *p* < 0.01, *** *p* < 0.001.

Regarding films light transmittance, Figure 1 shows that there were no significant differences (*p* > 0.05) between the two types of film in the UV spectrum range and none of the films allowed the passage of UV light as the percentages of transmittance was very low (from 0.03 to 1.81.,This means that the films are good barriers against UV radiation.

However, it was observed that the light transmittance capacity of the films started to be significantly different (*p* < 0.001) from 550 nm to 850 nm (visible light spectrum range), reaching higher values in films made with mucilage from whole seeds (82.12 vs. 66.27%). This decrease in light transmittance was also observed by Dick et al. [23] when they used chia flour instead of whole seeds. Moreover, these results were consistent with the differences in the opacity (Table 2) and could indicate that chia whole seed films will allow more light to pass through, which may lead to more oxidative reactions.

Comparing to other works, it was also observed that the light transmittance of the films made with defatted flour was close to those found in other chia films [36] and lower than other synthetic polymers films. However, in the whole seeds films, it was the opposite as their values were higher and closer to other synthetic films [39], as well as slightly higher than chia whole seeds films made with different concentrations of CM [18]. Therefore, it could be said that defatted flour films are better protective barriers against visible light than films made from whole seeds.

### 3.6. Mechanical Properties

**Figure 2:**
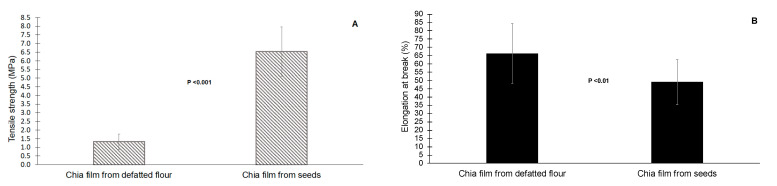
Tensile strength (**A**) and elongation at break (**B**) of the chia films made with whole seeds and defatted flour.

Tensile strength (TS) and elongation (E%) were evaluated to describe the relationship between the mechanical and chemical properties of the films. Tensile strength is defined as the maximum force (stress) and the elongation at break is the increase in the sample length until reaching the breakpoint [40]. Tensile strength and elongation at break are two fundamental properties that should be evaluated since films and packaging materials must be maintained their integrity during the processing, transport, and handling of the food that they are meant to protect. The results of mechanical properties of the films made with different chia matrixes (defatted flour and whole seeds) are illustrated in Figure 2 and there were significant differences (*p* < 0.001) in TS values of the films being higher in the films made with whole seeds (1.34 vs. 6.54 MPa). At the same time, it was observed that the E% increased significantly (*p* < 0.01) when we used defatted flour (from 49.17 to 66.23%). It was also noticed that the films made with whole seed did not recover their initial shape after deformation while films made with defatted flour remained practically intact after the test. Therefore, these findings mean that the films made with whole chia seeds are stronger but less elastic than films made with defatted flour. This increase in elongation and decrease in tensile strength was also observed by Dick et al. [18,23] when they used chia flour instead of whole seeds. This seems to indicate that the seeds grinding causes a release of cellular compounds (e.g., proteins) that could modify the mechanical properties of the films. Likewise, Salazar Vega et al. [29] and Muñoz et al. [28] have observed decreases in tensile strength when they increased the protein content of the films.

Overall, both of the films made with chia showed much lower TS values (1.34 to 6.54 vs. 16.56 to 25 MPa) and E% values up to 40% higher than other films made with different seeds composed of polysaccharides [18,20,23,24,26]. This meant that films made with chia might be a better choice for use as a coating material not subject to strong mechanical stress but requiring a high degree of flexibility. Edible coatings or films are characterized by being composed of food ingredients that are applied as a thin layer or by immersion in the foods [41]. Therefore, the low TS values along with the physical properties of both films confirm that they may be suitable raw materials for the development of food coatings but are not adequate as packaging material.

## 4. Conclusions

This work aimed to evaluate the feasibility of mucilage from defatted chia flour to form edible films as an alternative to the use of whole seeds. The physical, mechanical, and optical properties of the films were evaluated to determine their viability in edible film formation. Overall, it was observed that the films made with defatted flour were more elastic, better visible light barrier, less water-soluble, thicker, and less reddish than films made with whole seeds. Further studies are needed to evaluate in detail the effect of the mucilage composition on water vapor permeability and mechanical properties of the films as well as the compounds released after grinding the seeds to obtain the oil. The results obtained in this study demonstrated that the defatted flour from the seed oil extraction could be a useful by-product for developing edible coatings and films and a suitable option in the preservation of foods, thereby promoting the circular economy and reducing the amount of waste generated during chia oil production.

## Figures and Tables

**Table 1:** Properties of the chia films made with different matrixes (mean ± sd).

Parameters	Chia Film from Defatted Flour	Chia Film from Seeds	*p*-Value ^1^
% Moisture	22.44 ± 1.19	24.73 ± 1.83	*
% Water solubility	64.45 ± 3.90	82.56 ± 4.82	***
Thickness (mm)	0.12 ± 0.01	0.09 ± 0.01	***
WVP × 10^−10^ (g s^−1^ m^−1^ Pa^−1^)	0.58 ± 0.03	0.33 ± 0.03	***

^1^ Significance differences are indicated as follows: * *p* < 0.05; *** *p* < 0.001.

**Table 2:** Color parameters L*, a*, b*, opacity, and total color difference ∆E of the chia films made with different matrixes (mean ± sd).

Parameters	Chia Film from Defatted Flour	Chia Film from Seeds	*p*-Value ^1^
L*	56.42 ± 4.15	52.58 ± 2.67	NS
a*	5.45 ± 0.32	8.92 ± 0.72	***
b*	37.39 ± 4.16	38.98 ± 0.60	NS
∆E	54.4 ± 5.71	58.77 ± 2.12	NS
WI	42.31 ± 5.71	37.94 ± 2.12	NS
% Opacity	26.87 ± 0.75	25.21 ± 0.99	**

^1^ Significance differences are indicated as follows: NS: Not significant; ** *p* < 0.01; *** *p* < 0.001.

## Data Availability

The data presented in this study are available on request from the corresponding author.
